# Bis[bis­(3,5-diamino-1*H*-1,2,4-triazol-4-ium)copper(I)] tris­(hexa­fluoridosilicate)

**DOI:** 10.1107/S160053681004225X

**Published:** 2010-10-23

**Authors:** Marian Mys’kiv, Evgeny Goreshnik

**Affiliations:** a Department of Inorganic Chemistry, Ivan Franko National University, Cyryla & Mefodia, 6, L’viv, Ukraine; b Department of Inorganic Chemistry and Technology, Jožef Stefan Institute, Jamova 39 1000 Ljubljana, Slovenia

## Abstract

In the title compound, [Cu(C_2_H_6_N_5_)_2_]_2_(SiF_6_)_3_, the asymmetric unit is composed of one [Cu(H*L*)_2_]^3+^ cation (where *L* is 3,5-diamino-1,2,4-triazole) and one and a half SiF_6_^2−^ anions. The rather large positively charged guanazole ligand moiety promotes the low metal coordination number of 2 for the Cu^I^ atom. The compound was obtained using the electrochemical alternating-current technique starting from an ethanol–methanol solution of CuSiF_6_·4H_2_O and guanazole. In the crystal, N—H⋯F hydrogen bonds play an important role in the formation of a three-dimensional network. As a result of these hydrogen bonds, there are also π–π inter­actions [centroid–centroid distance = 3.3024 (14) Å] involving one of the triazole groups in mol­ecules related by an inversion center, and short Cu⋯N inter­actions [2.909 (3) Å] involving an –NH_2_ group, leading to the formation of a dimer-like arrangement.

## Related literature

For 1,2,4-triazole and its functionalized derivatives, see: Potts (1984 ▶). For complexes of the same ligand and copper(I) complexes of similar voluminous ligands, see: Aznar *et al.* (2006 ▶); Fabretti (1992 ▶); Goreshnik *et al.* (2004 ▶).
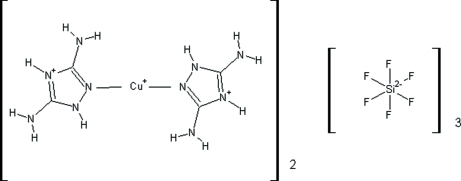


## Experimental

### 

#### Crystal data

[Cu(C_2_H_6_N_5_)_2_]_2_(SiF_6_)_3_
*M* *_r_* = 953.84
Triclinic, 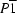

*a* = 7.482 (2) Å
*b* = 8.366 (1) Å
*c* = 12.131 (3) Å
α = 87.98 (2)°
β = 89.11 (2)°
γ = 67.89 (2)°
*V* = 703.1 (3) Å^3^
*Z* = 1
Mo *K*α radiation
μ = 1.81 mm^−1^
*T* = 293 K
0.24 × 0.20 × 0.04 mm

#### Data collection

Siemens AED2 diffractometer
Absorption correction: numerical (de Meulanaer & Tompa, 1965) ▶ *T*_min_ = 0.649, *T*_max_ = 0.935
4089 measured reflections
4089 independent reflections
3367 reflections with *I* > 2σ(*I*)
3 standard reflections every 60 min  intensity decay: 2%

#### Refinement

*R*[*F* ^2^ > 2σ(*F* ^2^)] = 0.053
*wR*(*F* ^2^) = 0.155
*S* = 1.06
4089 reflections
244 parameters
4 restraints
H atoms treated by a mixture of independent and constrained refinement
Δρ_max_ = 1.23 e Å^−3^
Δρ_min_ = −1.01 e Å^−3^

### 

Data collection: *STADI4* (Stoe & Cie, 1998 ▶); cell refinement: *STADI4*; data reduction: *X-RED* (Stoe & Cie, 1998 ▶); program(s) used to solve structure: *SHELXS86* (Sheldrick, 2008 ▶); program(s) used to refine structure: *SHELXL97* (Sheldrick, 2008 ▶); molecular graphics: *DIAMOND* (Crystal Impact, 2010 ▶), *ORTEPIII* (Burnett & Johnson, 1996 ▶) and *ORTEP-3* (Farrugia, 1997 ▶); software used to prepare material for publication: *enCIFer* (Allen *et al.*, 2004 ▶).

## Supplementary Material

Crystal structure: contains datablocks global, I. DOI: 10.1107/S160053681004225X/su2214sup1.cif

Structure factors: contains datablocks I. DOI: 10.1107/S160053681004225X/su2214Isup2.hkl

Additional supplementary materials:  crystallographic information; 3D view; checkCIF report

## Figures and Tables

**Table 1:** Hydrogen-bond geometry (Å, °)

*D*—H⋯*A*	*D*—H	H⋯*A*	*D*⋯*A*	*D*—H⋯*A*
N2—H2⋯F2^i^	0.86 (2)	1.86 (2)	2.694 (3)	166 (4)
N3—H3⋯F8	0.88 (2)	2.02 (3)	2.798 (3)	146 (4)
N4—H4*B*⋯F1^ii^	0.86	1.95	2.742 (4)	153
N4—H4*A*⋯F9^ii^	0.86	1.95	2.801 (3)	171
N5—H5*A*⋯F6^iii^	0.86	1.95	2.803 (3)	174
N5—H5*B*⋯F9^iv^	0.86	2.07	2.898 (3)	162
N7—H7⋯F4^i^	0.86 (2)	1.85 (2)	2.686 (3)	162 (4)
N8—H8⋯F7^v^	0.86 (2)	2.04 (3)	2.812 (3)	148 (4)
N8—H8⋯F3^v^	0.86 (2)	2.22 (3)	2.813 (3)	126 (3)
N9—H9*B*⋯F8^vi^	0.86	2.05	2.892 (3)	166
N9—H9*A*⋯F5^vii^	0.86	2.02	2.841 (4)	159
N10—H10*B*⋯F5^v^	0.86	2.22	2.909 (3)	137
N10—H10*A*⋯F6^iii^	0.86	2.02	2.845 (3)	160

Symmetry codes: (i) 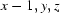; (ii) 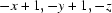; (iii) 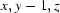; (iv) 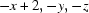; (v) 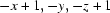; (vi) 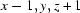; (vii) 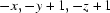.

**Fig. 1.**
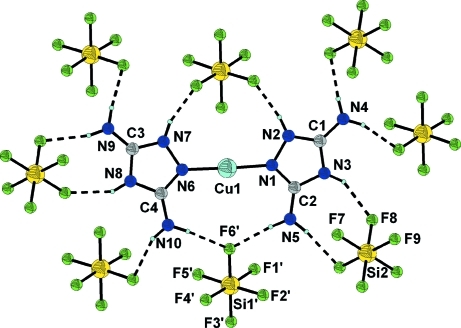
Copper surrounding of the title cation with displaceent ellipsoids drawn at the 50% probability level [Symmetry operation: (') = x, y-1, z].

**Fig. 2.**
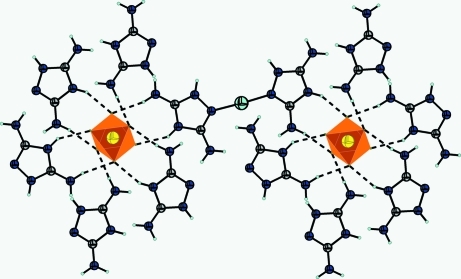
The environment of the SiF62- dianions in the title compound.

**Fig. 3.**
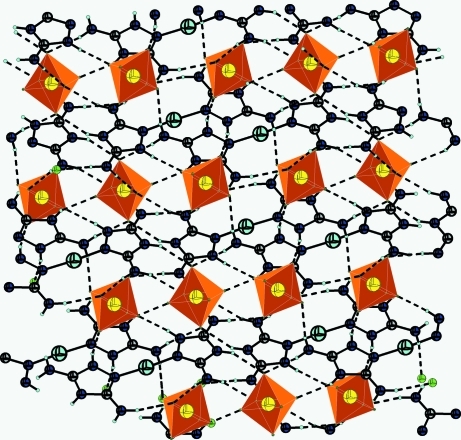
A view along the b-axis of the crystal packing of the title compound.

## supplementary crystallographic information

### Comment

1,2,4-triazole and its functionalized derivatives, particularly 3,5-diamino-1,2,4-triazole (*L*), have attracted great interest and are actively studied as ligands in the synthesis of coordination compounds, biologically active compounds with a wide range efficiency, and as components of high-energy compositions [Potts, 1984]. On the other hand only a few X-ray crystal structures of complexes of this triazole have been reported (Aznar *et al.*, 2006). The formation of low soluble polynuclear metal derivatives is one of the hindrances for structural studies of such compounds. It may be expected that the protonated form of the ligand (LH) will possess lower affinity to metal centers. Herein, we report on the synthesis and crystal structure of the title copper(I) hexafluorosilicate complex of LH.

Beside the positively charged state, the LH moiety demonstrates ability of metal coordination. In the structure of [Cu(LH)_2_]_2_(SiF_6_)_3_ each metal atom is bound to two nitrogen atoms from two LH moieties (Fig. 1). A similar linear copper(I) surrounding comprising of two nitrogen atoms from two voluminous ligand molecules was observed, for example, in the structure of bis(2-methylbenzimidazole)copper(I) dichlorocuprate(I) (Goreshnik *et al.*, 2004). Because of the low copper(I) ion coordination number both Cu–N distances appear to be rather short, 1.8747 (18) and 1.8749 (17) Å. Despite the cationic status of the ligand moiety the Cu - N bond length is practically the same [1.874 (2) Å] as in the above mentioned bis(2-methylbenzimidazole)copper(I) cation.

In the crystal each NH and NH_2 _hydrogen atom participates in the formation of strong N—H···F hydrogen bonds (Table 1). The closest NH_2_ group to the coordinated copper ion [Cu1···N10^i^ = 2.9092 (29) Å, symmetry code (i) = -*x* + 1, -*y*, -*z* + 1], forms noticeably shorter hydrogen bonds than all the others. Each of the two crystallographically independent SiF_6_^2-^ anions is bound to six LH units (Fig. 2). The [Cu(LH)]^3+^ and SiF_6_^2-^ units are interconnected by N—H···F bonds to form a three dimensional network (Fig. 3). In the crystal there are also π–π interactions involving triazole rings (N1—N3,C3,C4 = *Cg*2) related by an inversion center, with a centroid-to-centroid distance of 3.3024 (14)Å for *Cg*2···*Cg*2^ii^ [symmetry code (ii) = -*x*, -*y*, 1 - *z*].

As was already mentioned, the guanazolium moiety in this structure acts as a ligand despite its cationic status. Such behaviour was observed previously in the structure of platinum(II) dibromo bis(3,5-diamino-1(2)-triazolium) dibromide (Fabretti, 1992). It emphasizes the high affinity of this triazole derivative towards metal ions. The relatively large size of the LH units and their positive charge lead to the low coordination number of the copper ion.

### Experimental

The title compound was prepared using electrochemical synthesis. An ethanol solution of (LH)_2_SiF_6_ (where *L* = 3,5-diamino-1,2,4-triazole) was added to a solution of Cu_2_SiF_6_^.^4H_2_O (prepared by dissolving [(CuOH)_2_CO_3_] in H_2_SiF_6_) in CH_3_OH. This solution was then placed in a small test-tube and copper-wire electrodes were inserted. By usage of the alternating-current electrochemical technique at 0.5 V of tension during some days colourless crystals of the title compound appeared on the electrodes.

### Refinement

The N-bound H-atoms could all be located in difference Fourier maps. In the final cycles of least-squares refinement they were refined with distance restraints of 0.86 (2) Å with *U*_iso_(H) = 1.2*U*_eq_(N).

### Crystal data

**Table:**

[Cu(C_2_H_6_N_5_)_2_]_2_(SiF_6_)_3_	*Z* = 1
*M**_r_* = 953.84	*F*(000) = 474
Triclinic, *P*1	*D*_x_ = 2.253 Mg m^−^^3^
Hall symbol: -P 1	Mo *K*α radiation, λ = 0.71069 Å
*a* = 7.482 (2) Å	Cell parameters from 25 reflections
*b* = 8.366 (1) Å	θ = 35–45°
*c* = 12.131 (3) Å	µ = 1.81 mm^−^^1^
α = 87.98 (2)°	*T* = 293 K
β = 89.11 (2)°	Plate, colourless
γ = 67.89 (2)°	0.24 × 0.20 × 0.04 mm
*V* = 703.1 (3) Å^3^	

### Data collection

**Table:**

Siemens AED2 diffractometer	3367 reflections with *I* > 2σ(*I*)
Radiation source: fine-focus sealed tube	*R*_int_ = 0.0000
graphite	θ_max_ = 30.0°, θ_min_ = 1.7°
θ/2ω scans	*h* = −10→10
Absorption correction: numerical (de Meulanaer & Tompa, 1965)	*k* = −11→11
*T*_min_ = 0.649, *T*_max_ = 0.935	*l* = 0→17
4089 measured reflections	3 standard reflections every 60 min
4089 independent reflections	intensity decay: 2%

### Refinement

**Table:**

Refinement on *F*^2^	Primary atom site location: structure-invariant direct methods
Least-squares matrix: full	Secondary atom site location: difference Fourier map
*R*[*F*^2^ > 2σ(*F*^2^)] = 0.053	Hydrogen site location: inferred from neighbouring sites
*wR*(*F*^2^) = 0.155	H atoms treated by a mixture of independent and constrained refinement
*S* = 1.06	*w* = 1/[σ^2^(*F*_o_^2^) + (0.1004*P*)^2^ + 0.624*P*] where *P* = (*F*_o_^2^ + 2*F*_c_^2^)/3
4089 reflections	(Δ/σ)_max_ < 0.001
244 parameters	Δρ_max_ = 1.23 e Å^−^^3^
4 restraints	Δρ_min_ = −1.01 e Å^−^^3^

### Special details

**Table:**

Geometry. All e.s.d.'s (except the e.s.d. in the dihedral angle between two l.s. planes) are estimated using the full covariance matrix. The cell e.s.d.'s are taken into account individually in the estimation of e.s.d.'s in distances, angles and torsion angles; correlations between e.s.d.'s in cell parameters are only used when they are defined by crystal symmetry. An approximate (isotropic) treatment of cell e.s.d.'s is used for estimating e.s.d.'s involving l.s. planes.
Refinement. Refinement of *F*^2^ against ALL reflections. The weighted *R*-factor *wR* and goodness of fit *S* are based on *F*^2^, conventional *R*-factors *R* are based on *F*, with *F* set to zero for negative *F*^2^. The threshold expression of *F*^2^ > σ(*F*^2^) is used only for calculating *R*-factors(gt) *etc*. and is not relevant to the choice of reflections for refinement. *R*-factors based on *F*^2^ are statistically about twice as large as those based on *F*, and *R*- factors based on ALL data will be even larger.

### Fractional atomic coordinates and isotropic or equivalent isotropic displacement parameters (Å^2^)

**Table:**

	*x*	*y*	*z*	*U*_iso_*/*U*_eq_	
Cu1	0.23338 (6)	0.16548 (5)	0.35785 (3)	0.03274 (14)	
N1	0.2950 (4)	0.2228 (3)	0.2154 (2)	0.0266 (5)	
N2	0.1956 (4)	0.3827 (3)	0.1626 (2)	0.0286 (5)	
H2	0.084 (4)	0.453 (5)	0.182 (3)	0.034*	
N3	0.4365 (3)	0.2455 (3)	0.0583 (2)	0.0248 (5)	
H3	0.512 (5)	0.226 (5)	0.000 (2)	0.030*	
N4	0.2278 (5)	0.5262 (4)	−0.0040 (2)	0.0389 (7)	
H4A	0.1265	0.6165	0.0081	0.047*	
H4B	0.2942	0.5211	−0.0632	0.047*	
N5	0.5685 (4)	−0.0176 (3)	0.1625 (2)	0.0322 (6)	
H5A	0.5625	−0.0792	0.2197	0.039*	
H5B	0.6580	−0.0594	0.1143	0.039*	
N6	0.1586 (3)	0.1111 (3)	0.49796 (19)	0.0236 (4)	
N7	−0.0093 (4)	0.2215 (3)	0.54935 (19)	0.0245 (5)	
H7	−0.091 (5)	0.318 (3)	0.524 (3)	0.029*	
N8	0.1222 (3)	0.0061 (3)	0.66222 (19)	0.0238 (4)	
H8	0.132 (6)	−0.065 (4)	0.717 (2)	0.029*	
N9	−0.1716 (4)	0.2238 (4)	0.7183 (2)	0.0342 (6)	
H9A	−0.2647	0.3194	0.7016	0.041*	
H9B	−0.1717	0.1722	0.7809	0.041*	
N10	0.4006 (4)	−0.1545 (3)	0.5565 (2)	0.0296 (5)	
H10A	0.4684	−0.1618	0.4976	0.036*	
H10B	0.4393	−0.2340	0.6074	0.036*	
C1	0.2813 (4)	0.3948 (4)	0.0686 (2)	0.0257 (5)	
C2	0.4398 (4)	0.1416 (4)	0.1488 (2)	0.0226 (5)	
C3	−0.0290 (4)	0.1567 (4)	0.6484 (2)	0.0230 (5)	
C4	0.2343 (4)	−0.0189 (3)	0.5689 (2)	0.0215 (5)	
Si1	0.68995 (11)	0.55369 (9)	0.31802 (7)	0.02457 (18)	
F1	0.5751 (3)	0.5922 (3)	0.19565 (17)	0.0403 (5)	
F2	0.8718 (3)	0.5985 (3)	0.25878 (19)	0.0408 (5)	
F3	0.8049 (4)	0.3448 (3)	0.2924 (2)	0.0525 (6)	
F4	0.7987 (3)	0.5232 (3)	0.44239 (19)	0.0426 (5)	
F5	0.5036 (3)	0.5137 (3)	0.37463 (18)	0.0370 (4)	
F6	0.5785 (3)	0.7657 (2)	0.34582 (15)	0.0305 (4)	
Si2	1.0000	0.0000	0.0000	0.0211 (2)	
F7	0.9165 (3)	0.0973 (3)	0.11859 (16)	0.0347 (4)	
F8	0.7827 (3)	0.1080 (3)	−0.05936 (16)	0.0344 (4)	
F9	1.0741 (3)	0.1610 (2)	−0.03969 (18)	0.0344 (4)	

### Atomic displacement parameters (Å^2^)

**Table:**

	*U*^11^	*U*^22^	*U*^33^	*U*^12^	*U*^13^	*U*^23^
Cu1	0.0426 (2)	0.0340 (2)	0.0220 (2)	−0.01538 (17)	0.01182 (15)	0.00019 (14)
N1	0.0254 (11)	0.0263 (11)	0.0219 (11)	−0.0033 (9)	0.0087 (8)	0.0016 (8)
N2	0.0266 (11)	0.0262 (11)	0.0235 (11)	0.0004 (9)	0.0108 (9)	−0.0012 (9)
N3	0.0242 (10)	0.0222 (10)	0.0244 (11)	−0.0048 (9)	0.0127 (8)	−0.0032 (8)
N4	0.0456 (16)	0.0234 (12)	0.0305 (13)	0.0056 (11)	0.0124 (11)	0.0047 (10)
N5	0.0250 (11)	0.0271 (12)	0.0372 (14)	−0.0022 (9)	0.0096 (10)	0.0048 (10)
N6	0.0254 (10)	0.0238 (10)	0.0204 (10)	−0.0081 (8)	0.0065 (8)	−0.0014 (8)
N7	0.0259 (11)	0.0228 (10)	0.0208 (10)	−0.0049 (9)	0.0040 (8)	0.0014 (8)
N8	0.0225 (10)	0.0276 (11)	0.0200 (10)	−0.0084 (9)	0.0051 (8)	0.0010 (8)
N9	0.0238 (11)	0.0422 (15)	0.0272 (12)	−0.0021 (10)	0.0094 (9)	0.0018 (10)
N10	0.0252 (11)	0.0258 (12)	0.0319 (13)	−0.0033 (9)	0.0076 (9)	−0.0002 (9)
C1	0.0280 (13)	0.0222 (12)	0.0223 (12)	−0.0041 (10)	0.0100 (10)	−0.0043 (9)
C2	0.0194 (11)	0.0240 (12)	0.0219 (12)	−0.0057 (9)	0.0071 (9)	−0.0012 (9)
C3	0.0209 (11)	0.0275 (13)	0.0204 (11)	−0.0090 (10)	0.0036 (9)	−0.0012 (9)
C4	0.0230 (11)	0.0223 (11)	0.0204 (11)	−0.0102 (9)	0.0042 (9)	−0.0006 (9)
Si1	0.0230 (3)	0.0170 (3)	0.0284 (4)	−0.0017 (3)	0.0092 (3)	−0.0018 (3)
F1	0.0443 (11)	0.0473 (12)	0.0260 (9)	−0.0130 (9)	0.0055 (8)	−0.0072 (8)
F2	0.0294 (9)	0.0356 (10)	0.0544 (13)	−0.0091 (8)	0.0225 (9)	−0.0072 (9)
F3	0.0511 (13)	0.0212 (9)	0.0772 (17)	−0.0041 (9)	0.0209 (12)	−0.0134 (10)
F4	0.0439 (11)	0.0338 (10)	0.0439 (12)	−0.0083 (9)	−0.0108 (9)	0.0122 (9)
F5	0.0336 (9)	0.0348 (10)	0.0425 (11)	−0.0134 (8)	0.0122 (8)	0.0030 (8)
F6	0.0348 (9)	0.0183 (7)	0.0297 (9)	−0.0005 (6)	0.0104 (7)	−0.0020 (6)
Si2	0.0195 (4)	0.0196 (4)	0.0209 (5)	−0.0037 (3)	0.0087 (3)	−0.0007 (3)
F7	0.0376 (10)	0.0352 (10)	0.0269 (9)	−0.0087 (8)	0.0158 (7)	−0.0087 (7)
F8	0.0236 (8)	0.0379 (10)	0.0326 (9)	−0.0019 (7)	0.0047 (7)	0.0052 (8)
F9	0.0327 (9)	0.0246 (8)	0.0454 (11)	−0.0108 (7)	0.0164 (8)	−0.0009 (7)

### Geometric parameters (Å, °)

**Table:**

Cu1—N1	1.874 (2)	N8—C4	1.372 (3)
Cu1—N6	1.875 (2)	N8—H8	0.864 (19)
N1—C2	1.321 (3)	N9—C3	1.315 (3)
N1—N2	1.399 (4)	N9—H9A	0.8600
N2—C1	1.320 (3)	N9—H9B	0.8600
N2—H2	0.856 (19)	N10—C4	1.342 (4)
N3—C1	1.356 (3)	N10—H10A	0.8600
N3—C2	1.371 (4)	N10—H10B	0.8600
N3—H3	0.877 (19)	Si1—F3	1.670 (2)
N4—C1	1.324 (4)	Si1—F5	1.683 (2)
N4—H4A	0.8600	Si1—F1	1.686 (2)
N4—H4B	0.8600	Si1—F2	1.686 (2)
N5—C2	1.324 (4)	Si1—F4	1.691 (2)
N5—H5A	0.8600	Si1—F6	1.6959 (19)
N5—H5B	0.8600	Si2—F7^i^	1.6716 (18)
N6—C4	1.316 (4)	Si2—F7	1.6716 (18)
N6—N7	1.401 (3)	Si2—F9^i^	1.6912 (18)
N7—C3	1.329 (4)	Si2—F9	1.6912 (18)
N7—H7	0.863 (19)	Si2—F8	1.6920 (19)
N8—C3	1.347 (4)	Si2—F8^i^	1.6920 (19)
			
N1—Cu1—N6	177.04 (11)	N9—C3—N7	126.7 (3)
C2—N1—N2	105.2 (2)	N9—C3—N8	126.3 (3)
C2—N1—Cu1	131.5 (2)	N7—C3—N8	106.9 (2)
N2—N1—Cu1	122.95 (18)	N6—C4—N10	126.3 (2)
C1—N2—N1	110.1 (2)	N6—C4—N8	110.1 (2)
C1—N2—H2	125 (3)	N10—C4—N8	123.5 (3)
N1—N2—H2	124 (3)	F3—Si1—F5	90.85 (12)
C1—N3—C2	107.6 (2)	F3—Si1—F1	91.99 (14)
C1—N3—H3	122 (3)	F5—Si1—F1	88.81 (12)
C2—N3—H3	131 (3)	F3—Si1—F2	90.24 (12)
C1—N4—H4A	120.0	F5—Si1—F2	178.23 (12)
C1—N4—H4B	120.0	F1—Si1—F2	89.76 (12)
H4A—N4—H4B	120.0	F3—Si1—F4	90.31 (14)
C2—N5—H5A	120.0	F5—Si1—F4	90.60 (12)
C2—N5—H5B	120.0	F1—Si1—F4	177.64 (12)
H5A—N5—H5B	120.0	F2—Si1—F4	90.79 (13)
C4—N6—N7	104.9 (2)	F3—Si1—F6	178.49 (13)
C4—N6—Cu1	133.6 (2)	F5—Si1—F6	90.04 (10)
N7—N6—Cu1	121.44 (18)	F1—Si1—F6	89.24 (11)
C3—N7—N6	110.1 (2)	F2—Si1—F6	88.90 (11)
C3—N7—H7	122 (3)	F4—Si1—F6	88.47 (11)
N6—N7—H7	128 (3)	F7^i^—Si2—F7	180.00 (15)
C3—N8—C4	107.9 (2)	F7^i^—Si2—F9^i^	90.37 (10)
C3—N8—H8	123 (3)	F7—Si2—F9^i^	89.63 (10)
C4—N8—H8	129 (3)	F7^i^—Si2—F9	89.63 (10)
C3—N9—H9A	120.0	F7—Si2—F9	90.37 (10)
C3—N9—H9B	120.0	F9^i^—Si2—F9	180.00 (16)
H9A—N9—H9B	120.0	F7^i^—Si2—F8	90.01 (10)
C4—N10—H10A	120.0	F7—Si2—F8	89.99 (10)
C4—N10—H10B	120.0	F9^i^—Si2—F8	89.66 (10)
H10A—N10—H10B	120.0	F9—Si2—F8	90.34 (10)
N2—C1—N4	127.3 (3)	F7^i^—Si2—F8^i^	89.99 (10)
N2—C1—N3	107.2 (3)	F7—Si2—F8^i^	90.01 (10)
N4—C1—N3	125.4 (3)	F9^i^—Si2—F8^i^	90.34 (10)
N1—C2—N5	126.8 (3)	F9—Si2—F8^i^	89.66 (10)
N1—C2—N3	109.8 (2)	F8—Si2—F8^i^	180.00 (12)
N5—C2—N3	123.3 (2)		
			
N6—Cu1—N1—C2	−133 (2)	N2—N1—C2—N3	1.2 (3)
N6—Cu1—N1—N2	54 (2)	Cu1—N1—C2—N3	−172.2 (2)
C2—N1—N2—C1	−0.6 (3)	C1—N3—C2—N1	−1.3 (3)
Cu1—N1—N2—C1	173.4 (2)	C1—N3—C2—N5	176.9 (3)
N1—Cu1—N6—C4	135 (2)	N6—N7—C3—N9	−178.5 (3)
N1—Cu1—N6—N7	−46 (2)	N6—N7—C3—N8	−0.3 (3)
C4—N6—N7—C3	0.5 (3)	C4—N8—C3—N9	178.2 (3)
Cu1—N6—N7—C3	−178.76 (19)	C4—N8—C3—N7	0.0 (3)
N1—N2—C1—N4	179.3 (3)	N7—N6—C4—N10	−177.9 (3)
N1—N2—C1—N3	−0.2 (4)	Cu1—N6—C4—N10	1.2 (5)
C2—N3—C1—N2	0.9 (3)	N7—N6—C4—N8	−0.5 (3)
C2—N3—C1—N4	−178.6 (3)	Cu1—N6—C4—N8	178.6 (2)
N2—N1—C2—N5	−177.0 (3)	C3—N8—C4—N6	0.3 (3)
Cu1—N1—C2—N5	9.7 (5)	C3—N8—C4—N10	177.8 (3)

Symmetry codes: (i) −*x*+2, −*y*, −*z*.

### Hydrogen-bond geometry (Å, °)

**Table:**

*D*—H···*A*	*D*—H	H···*A*	*D*···*A*	*D*—H···*A*
N2—H2···F2^ii^	0.86 (2)	1.86 (2)	2.694 (3)	166 (4)
N3—H3···F8	0.88 (2)	2.02 (3)	2.798 (3)	146 (4)
N4—H4B···F1^iii^	0.86	1.95	2.742 (4)	153
N4—H4A···F9^iii^	0.86	1.95	2.801 (3)	171
N5—H5A···F6^iv^	0.86	1.95	2.803 (3)	174
N5—H5B···F9^i^	0.86	2.07	2.898 (3)	162
N7—H7···F4^ii^	0.86 (2)	1.85 (2)	2.686 (3)	162 (4)
N8—H8···F7^v^	0.86 (2)	2.04 (3)	2.812 (3)	148 (4)
N8—H8···F3^v^	0.86 (2)	2.22 (3)	2.813 (3)	126 (3)
N9—H9B···F8^vi^	0.86	2.05	2.892 (3)	166
N9—H9A···F5^vii^	0.86	2.02	2.841 (4)	159
N10—H10B···F5^v^	0.86	2.22	2.909 (3)	137
N10—H10A···F6^iv^	0.86	2.02	2.845 (3)	160

Symmetry codes: (ii) *x*−1, *y*, *z*; (iii) −*x*+1, −*y*+1, −*z*; (iv) *x*, *y*−1, *z*; (i) −*x*+2, −*y*, −*z*; (v) −*x*+1, −*y*, −*z*+1; (vi) *x*−1, *y*, *z*+1; (vii) −*x*, −*y*+1, −*z*+1.

## References

[bb1] Allen, F. H., Johnson, O., Shields, G. P., Smith, B. R. & Towler, M. (2004). *J. Appl. Cryst.* **37**, 335–338.

[bb2] Aznar, E., Ferrer, S., Borrás, J., Lloret, F., Liu-González, M., Rodríguez-Prieto, M. & García-Granda, S. (2006). *Eur. J. Inorg. Chem.* pp. 5115–5125.

[bb3] Burnett, M. N. & Johnson, C. K. (1996). *ORTEPIII* Report ORNL-6895. Oak Ridge National Laboratory, Tennessee, USA.

[bb4] Crystal Impact (2010). *DIAMOND* Crystal Impact GbR, Bonn, Germany

[bb5] Fabretti, A. C. (1992). *J. Crystallogr. Spectrosc. Res.* **22**, 523–526.

[bb6] Farrugia, L. J. (1997). *J. Appl. Cryst.* **30**, 565.

[bb7] Goreshnik, E., Schollmeyer, D. & Mys’kiv, M. (2004). *Acta Cryst.* E**60**, m279–m281. 10.1107/S0108270105000806 15750226

[bb8] Meulenaer, J. de & Tompa, H. (1965). *Acta Cryst.* **19**, 1014–1018.

[bb9] Potts, K. T. (1984). Editor. *Comprehensive Heterocycle Chemistry*, Vol. 5. Oxford: Pergamon Press.

[bb10] Sheldrick, G. M. (2008). *Acta Cryst.* A**64**, 112–122. 10.1107/S0108767307043930 18156677

[bb11] Stoe & Cie (1998). *STADI4* and *X-RED* Stoe &Cie GmbH, Darmstadt, Germany.

